# Connecting Circuits with Networks in Addiction Neuroscience: A Salience Network Perspective

**DOI:** 10.3390/ijms24109083

**Published:** 2023-05-22

**Authors:** Adriana K. Cushnie, Wei Tang, Sarah R. Heilbronner

**Affiliations:** 1 Department of Neuroscience, University of Minnesota Twin Cities, 2-164 Jackson Hall, 321 Church St. SE, Minneapolis, MN 55455, USA; cushn003@umn.edu; 2 Department of Computer Science, Indiana University Bloomington, Bloomington, IN 47408, USA; 3 Department of Neurosurgery, Baylor College of Medicine, Houston, TX 77030, USA

**Keywords:** salience network, default mode network, addiction, insula, cingulate cortex

## Abstract

Human neuroimaging has demonstrated the existence of large-scale functional networks in the cerebral cortex consisting of topographically distant brain regions with functionally correlated activity. The salience network (SN), which is involved in detecting salient stimuli and mediating inter-network communication, is a crucial functional network that is disrupted in addiction. Individuals with addiction display dysfunctional structural and functional connectivity of the SN. Furthermore, while there is a growing body of evidence regarding the SN, addiction, and the relationship between the two, there are still many unknowns, and there are fundamental limitations to human neuroimaging studies. At the same time, advances in molecular and systems neuroscience techniques allow researchers to manipulate neural circuits in nonhuman animals with increasing precision. Here, we describe attempts to translate human functional networks to nonhuman animals to uncover circuit-level mechanisms. To do this, we review the structural and functional connections of the salience network and its homology across species. We then describe the existing literature in which circuit-specific perturbation of the SN sheds light on how functional cortical networks operate, both within and outside the context of addiction. Finally, we highlight key outstanding opportunities for mechanistic studies of the SN.

## 1. Introduction

For human neuroscience, functional neuroimaging has allowed the study of the human brain in terms of regional and network properties. Functional magnetic resonance imaging (fMRI), in particular, allows for whole-brain investigations of correlated activity across brain regions that may be functionally related but physically distant. At the same time, nonhuman animal studies have a long tradition of manipulating and measuring specific neuronal circuits and cell populations. Such studies are aided by invasive molecular techniques that are not feasible in human experimental studies, including chemogenetics and optogenetics. In this review, we will aim to bridge the gap between the human network and nonhuman animal circuit and molecular approaches, with a particular focus on the salience network in addiction.

Intrinsic functional connectivity networks are sets of brain regions that may be spatially dispersed but display temporally correlated spontaneous neural activity across time, independent of external stimuli [1,2]. Several networks commonly emerge from neuroimaging studies, all of which are relatively stable and reproducible across sessions and individuals [3,4,5,6,7], although there is also significant inter-individual variability in the precise locations of the constitutive regions, [8,9,10]. In many cases, the activity of intrinsic networks at rest is correlated with performance during task-based fMRI [11,12,13]. Furthermore, although these networks have been primarily identified using resting-state functional connectivity, the constitutive regions also often co-activate during tasks. Increasingly, we expect that the characterization of intrinsic functional connectivity networks will be vital to identifying and manipulating healthy and aberrant brain functioning [14,15,16].

Several networks show abnormal intra- and inter-network functional connectivity in addiction [17,18,19,20,21]. These include the default mode network (DMN), a set of brain regions that are functionally connected at rest and are characterized by increased activity at rest and during internally directed processes such as autobiographical memory [22,23,24,25], the central executive network (CEN), which is most active when engaging in task and goal-directed behavior [26,27,28], and the salience network (SN), a set of brain regions involved in identifying salient stimuli and incorporating this information to inform decision making [3,29,30]. The SN is also a controller of the DMN and CEN, situating it at the center of a ‘triple network’ model of brain functioning [26,31,32,33,34,35]. Here, we will focus on the SN because of this central role, its importance in addiction, its cross-species relevance, and because it has not yet been the subject of a similarly focused review.

The SN as a unified entity was initially defined using resting-state human functional magnetic resonance imaging (rs-fMRI), during which participants were only required to remain still [3]. The high functional connectivity among SN regions during rest results from their correlated blood-oxygen-level-dependent (BOLD) activity. In addition, the subregions of the SN often co-activate under certain task demands, for example, when engaging in tasks that require cognitive flexibility (e.g., the Trail Making Test, Go/No Go task, and Stroop task [3,36]). The SN core cortical nodes are the anterior insular cortex (AIC) and the dorsal anterior cingulate cortex (dACC). (See below for discussion of additional potential SN regions.) Human intracranial recordings have also revealed that the AIC and dACC are more functionally connected electrophysiologically to one another than they are to other (non-SN) regions [37].

The SN detects the most salient stimuli (a salient stimulus can be novel, important, or attention-commanding) among competing external and internal cues, and it does so irrespective of valence. The SN then orients cognitive resources appropriately [3,38,39,40]. Because of its role in general salience detection, the SN unifies information regarding conflict monitoring, interoception, autonomic signals, homeostasis, reward information, and emotion processing [30,41,42,43,44].

Additionally, the SN plays a crucial role in coordinating the switch in functional activity across multiple networks [29,45,46,47]. The SN, therefore, sits at the center of what has become known as the ‘triple network’ (DMN, CEN, and SN). In this model, the DMN directs cognitive resources (such as attention) internally, the CEN directs cognitive resources externally, and the SN detects salient events to direct the functioning of the DMN vs. CEN. Invasive recordings in humans suggest that SN regions help to control DMN and CEN activity [37]. There is even causal evidence of this triple network model from human patients with traumatic brain injury: SN damage impairs DMN connectivity and cognitive control [48]. 

## 2. Functions and Connectivity of the Nodes of the Salience Network

The main nodes of the SN—the anterior insular cortex (AIC) and the dorsal anterior cingulate cortex (dACC)—have individually been the subjects of intensive study. The anatomy and functions of the SN, as well as of its individual component regions, have also been the subject of excellent, recent reviews, and we will not try to completely cover the nuances of these topics here [49,50,51,52,53,54,55,56,57]. Instead, below, we briefly describe the anatomy and functions of these two regions. Here, it is worth noting that our knowledge of the anatomical connections of the AIC and dACC are necessarily derived from nonhuman animal models (particularly nonhuman primates) [58], and as discussed in more detail below, the homology with human brain structures may be imperfect.

### 2.1. The Anterior Insular Cortex (AIC)

#### 2.1.1. Anatomy of the AIC

The insular cortex is located beneath the lateral sulcus of the Sylvian fissure and is covered by the frontoparietal operculum and superior temporal cortex (in humans and nonhuman primates—in rodents, the insular cortex is exposed on the lateral surface) [59,60]. The insular cortex contains three large subdivisions: anterior, middle, and posterior [53,59,61,62,63,64,65,66,67,68,69]. Briefly, the anterior region of the insular cortex (which is the region that is part of the SN), which has both dorsal dysgranular and ventral agranular zones, has prominent connections (at least in the nonhuman primate) with the orbitofrontal regions, anterior cingulate regions, anterior temporal regions, and the olfactory cortex [70]. Along with part of the frontal operculum, the anterior insula also contains the primary gustatory cortex (in nonhuman primates, but is more medial in humans), with projections from thalamic neurons coding for taste information [70,71]. The posterior insula, which is granular, contains prominent connections to the mid-posterior cingulate cortex, somatosensory, posterior temporal regions, supplementary motor area, and the parietal lobe [50,62,72,73]. The middle insular cortex, which is dysgranular, displays a mix of connections that is typical for the anterior and posterior insular divisions [74,75]. The anterior to posterior axis of the insula is present not only in cytoarchitecture, connectivity, and functions, but also in electrophysiological properties such as oscillations [76].

The dorsal dysgranular and ventral agranular zones of the AIC themselves have different connectivity profiles. The dorsal dysgranular AIC connects with the rostral dorsolateral prefrontal cortex, dACC, precentral operculum, rostral inferior parietal cortex, anterior inferior frontal gyrus, dorsal anterior temporal lobe, and dorsal striatum [62,63,72,74,77,78,79,80]. By comparison, the ventral agranular AIC connects to the pregenual anterior cingulate cortex, dACC, lateral orbitofrontal cortex, amygdala, ventral striatum, substantia nigra, and ventral tegmental area [77,81,82,83,84,85]. These connections are likely important for affect-related processing, and these target regions are often implicated in addiction. The ventral agranular AIC also contains von Economo neurons and Fork cells. Von Economo cells are large “spindle cells” with a single basal dendrite that are thought to be crucial for the rapid transmission of information over long distances [86,87]. Fork neurons have distinct morphology, a divided apical dendrite and are typically found along with von Economo neurons [87,88]. Interestingly, it has been proposed that von Economo cells facilitate the signaling that supports the SN [29,45,88].

#### 2.1.2. Functions of the AIC

Numerous functions, including emotional processing, pain processing, cognition (including language), and maintaining a homeostatic balance, have been attributed to the AIC [6,42,60,62,89,90,91,92,93]. The AIC, the ventral striatum, and the amygdala are consistently co-activated during neuroimaging studies of general emotional processing [94,95,96]. Similarly, the AIC, along with the ACC, is involved in the motivational-affective (as opposed to sensory) aspects of pain [97,98], including seeing other people in pain [91,99]. The AIC’s involvement in emotion and pain processing extends to empathy: it encodes others’ emotional states [100,101], potentially as they relate to the affective states that others’ emotions can induce, and potentially because of the decision-making that others’ emotions may require [91].

The insular cortex is the established region for viscerosensory processing; it is the part of the brain that encodes body temperature, visceral sensation, and arousal state [41,42,102,103,104,105]. The AIC is also important for interoception, which involves integrating internal visceral and autonomic changes with salient external stimuli to maintain homeostasis and inform subsequent behaviors [42,106,107]. In particular, the AIC compares current states with previous conditions to update information and guide behaviors. This may be why the AIC is frequently activated in response to novel stimuli [38]: the need to stay up-to-date about the current interoceptive state means that the AIC finds novel stimuli, particularly within its functional wheelhouse.

Furthermore, the AIC is essential in mediating the switch between a restful and internally focused state (driven by the activity in the DMN) and engaging in tasks (CEN and SN) [45,90]. The AIC displays activity before the other nodes in the SN (based on latency analysis), such that it has a strong and causal effect on the activity of the dACC and leads to an increase in intra-network connectivity [23,45,108]. This occurs before the inactivation of the DMN and simultaneous activation of the CEN when transitioning to a task/goal-oriented state. This transition is facilitated by the connections of the AIC with nodes in the DMN and CEN [29,33,45,102].

### 2.2. Dorsal Anterior Cingulate Cortex (dACC)

#### 2.2.1. Anatomy of dACC

The anterior cingulate cortex (ACC) is situated rostrally in the medial wall of each hemisphere [109]. The ACC has heterogeneous cytoarchitectural and connectivity patterns [81,82,85,110]. It is composed of at least three subdivisions: area 25 is situated beneath the corpus callosum, area 32 is rostral, extending around the genus of the corpus callosum (and, in humans, extending dorsally to area 24), and area 24 is dorsal to the rostral corpus callosum. The dorsal ACC (dACC, area 24 in nonhuman primates, and 24 and dorsal 32 in humans [111]) is the part of the ACC most consistently implicated in the SN.

The dACC connects with an intriguing mix of limbic, cognitive, and motor brain regions. These include the posterior cingulate cortex, amygdala, hypothalamus, dorsolateral prefrontal cortex, orbitofrontal cortex, both the ventral and dorsal striatum, inferior temporal lobe, and multisensory temporal cortical regions. In addition, the dACC also contains cingulate motor areas (CMAs) (called cingulate motor zones in nonhuman primates) [112,113]. These regions have direct projections to premotor areas, the motor cortex, and even the spinal cord. Generally, connections with emotion-related regions are concentrated more rostrally in the dACC, whereas connections with motor-related regions are concentrated more caudally in the dACC, and connections with cognitive structures are strongest in between [82,85,110,114].

#### 2.2.2. Functions of dACC

The dACC, similar to the AIC, has been implicated in many cognitive and emotional functions. Chief among these is decision-making and pain processing. During reward-guided decision-making, the dACC monitors rewards, errors, and conflict [84,115,116,117]. This may be in service of future movements, such that dACC can facilitate changes in actions, such as promoting alternative choices in subsequent trials [118,119,120,121]. Pain processing is focal in the ventral subregion of the dACC [122,123,124], the stimulation of which evokes the will to persevere [125]. Such findings reveal mechanisms of cognitive control over physical and psychological pain [126,127]. The dACC’s role in cognitive control extends to negative emotion and affect via connections with the amygdala and ventral striatum [128,129].

### 2.3. Additional Regions

Although we have focused here on the dACC and AIC, other brain regions are sometimes assigned to the SN, including the paracingulate, ventrolateral prefrontal, superior temporal, opercular, supplementary motor, and pre-supplementary motor cortices [3,130]. Subcortically, it is often associated with the amygdala, mediodorsal thalamus, hypothalamus, periaqueductal gray, substantia nigra, and ventral tegmental area [3,30]. Confusingly, SN regions can be functionally connected and/or co-active with brain regions canonically belonging to other networks, particularly in the dorsolateral prefrontal cortex, which is usually assigned to the CEN [3]. Perhaps this is due to triple network interactions across networks (although such an explanation does raise the problem of how, precisely, the SN is defined). In general, these issues are not unique to the SN; the interacting nature of these brain networks means that definitions may vary according to the measurement used, and while core regions (the dACC and AIC) remain consistent, others fluctuate across contexts and studies.

### 2.4. Relationship with the Cingulo-Opercular Network

The SN seemingly overlaps with the cingulo-opercular network (CON) [131,132]. The CON was identified in human fMRI studies using a region of interest (ROI) approach along with graph theory metrics to identify groups of regions that display functional connectivity [131,132]. This analysis identified the CON as consisting of two core regions—the dACC and dAIC/frontal operculum—along with other prefrontal and parietal regions implicated in various control functions [131,132]. Functionally, the CON is active in tasks requiring executive control, particularly when establishing and maintaining task sets and tonic alertness [132,133,134,135,136].

The first papers detailing the SN and CON were released at roughly the same time [3,131,132]. While the two networks were initially viewed as identical, further studies suggest a possible distinction in the locations of their core nodes: the CON may be anchored more dorsally and the SN more ventrally in the AIC [36,54,79,132,134,137,138]. Unsurprisingly, because of the similarities between the networks, there are instances in which the names CON and SN are used interchangeably or lumped together [6,139,140].

Similarities and differences between the CON and the SN are yet to be established. There is a possibility that the SN’s core nodes dynamically interact with different cortical regions to accomplish different tasks based on specific cognitive demands. To resolve this issue, studies that go beyond solely examining the functional imaging literature but instead also explore the anatomical basis of these networks will be valuable. For example, as described above, anatomical connectivity and cytoarchitecture differentiate the dorsal vs. ventral AIC, which could also support distinct functional connectivity. Moreover, some of these subregions may be more or less well represented in nonhuman animal species used for preclinical studies. The cross-species study of the SN by Tsai and colleagues (2020) (discussed in detail below) provides an excellent example of this approach [141].

## 3. The Salience Network in Addiction

Decades of human neuroimaging work have not only demonstrated that the SN is abnormal in addiction but have coalesced around many of the specifics of its pathophysiology. First, the SN is structurally altered in addiction; specifically, evidence points to reductions in volume, gray matter density, and white matter integrity in regions of the SN in addiction [107,142,143,144,145,146,147,148]. For example, alcohol use disorder and nicotine addiction are associated with a reduction in gray matter density in the AIC and ACC [20,143,149,150], as well as reduced cerebral blood flow to the AIC [149,151,152,153]. Interestingly, there is a reduced density of VENs in the AIC of individuals with alcohol dependency compared with controls [154]. Furthermore, reduced AIC volume correlates with higher levels of impulsivity and compulsivity in individuals with alcohol dependency [20]. Similar findings have also been identified for individuals with cocaine dependency [155,156]. Additionally, AIC cortical volume is negatively associated with the severity of alcohol dependency symptoms; thus, there is reduced volume of the AIC and ACC in alcohol dependency [20]. Furthermore, aberrant white matter fractional anisotropy (a measure from diffusion MRI used as a proxy for white matter integrity) found in the cingulum bundle, which is adjacent to the dACC, is correlated with alcohol, heroin, and cocaine use [157,158,159]. Notably, the SN is not *solely* involved in addiction: several different psychiatric diagnoses (including addiction) are associated with reduced gray matter density in the dACC and the AIC [160].

Second, the individual nodes of the SN are functionally disrupted in addiction. In general, in the SN, addiction seems to be associated with reduced activity and connectivity in response to non-drug, salient stimuli but with enhanced activity and connectivity in response to drug-related stimuli, although activity can be differentially modulated according to the drug and current state [161,162,163,164]. For instance, methamphetamine dependence is associated with reduced activity in the AIC while performing decision-making tasks [165,166] and when viewing negative, non-drug stimuli [167]. Blunted insula activity is also observed in adolescent light smokers viewing pleasurable food [168]. Increased AIC and dACC activity while viewing smoking cues predicts relapse, and attention was biased towards smoking-related cues [169,170,171]. Augmented insular reactivity to smoking cues is also associated with increased activity in the SN [170]. Similarly, dACC activity increases with self-reported drug craving [172]. Likewise, coherent activity in the dACC is negatively associated with decision latency in alcohol use disorder [173]. Diminished activity in the dACC is observed in cocaine users; furthermore, the amount of substance used is negatively correlated with activity in this region [174,175,176]. Moreover, heroin addiction is associated with elevated functional coupling between the dACC and the ventral striatum [177]. The alteration in functional activity is associated with disrupted functions, such as disrupted decision-making, strong associations between drugs of abuse and reward, interoception, and emotion regulation [107,163,178,179].

The SN also interacts with other distributed networks to create the aberrant cognition and behaviors observable in addiction. During nicotine deprivation, there is an increase in DMN activity; thus, attention is thought to be internally focused, which is often associated with withdrawal [180]. Likewise, abstinent heroin and cocaine users display elevated functional connectivity between the insular cortex and amygdala [181,182]. Conversely, during nicotine administration, the SN mediates an increase in CEN activity, allowing for engagement in self-administration [17,183,184]. This implies that SN dysfunction, which would result in aberrant switching between the CEN and DMN, could be crucial in addiction formation and maintenance by potentially producing the hyper/hypo activation of one network (DMN vs. CEN) relative to the other.

Finally, there is some causal evidence of the role of the SN in addiction in humans. Smokers who have sustained damage to the insular cortex display a reduction in addictive smoking behaviors and are more likely to quit smoking than smokers who sustained damage to other brain areas and have reduced propensity for relapse [142,185,186]. This fits the observation that the insular cortex has reduced activity during abstinence [145]. Furthermore, damage to the insular cortex has been linked to a decrease in nicotine withdrawal symptoms [187]. Smoking addiction was disrupted following lesions to brain regions that display positive functional connectivity with the dorsal cingulate cortex, lateral prefrontal cortex, and insula cortex [188]. Interestingly, when insula damage is combined with basal ganglia damage, the effects tend to be stronger, with a higher propensity for quitting smoking [189]. The strong, consistent finding that lesions to the insula disrupt addiction sits in apparent contradiction to the equally strong finding that addiction is associated with reduced gray matter volumes (see above) [163]. This discrepancy has not yet been resolved, but may, on further study, be explained by the different cognitive, emotional, and interoceptive processes necessary to cease drug use permanently vs. those induced by continued drug use vs. those at play in individuals who have never abused drugs. Another possibility is that different subregions of the insula (or the overlying white matter) may be responsible for lesion vs. volumetric effects, or that the efficacy of insular signaling may be affected. Although further research is necessary to resolve these issues, the different types of studies do consistently point to the AIC as central to addiction. 

There is also evidence that the SN interacts with the brain’s dopaminergic system, which is critical, not only for identifying environmental stimuli that are behaviorally relevant, signaling reward prediction errors, and responding to surprising stimuli [190,191], but also for developing and maintaining addiction. Drugs of abuse typically lead to an increase in dopamine activity [21,192,193,194,195,196,197,198], and after repeated use of drugs, basal levels of dopamine decrease below those in the pre-drug state. Additionally, during short-term abstinence from drugs, dopamine activity is also depreciated [178,199,200]. These dopaminergic effects have complex relationships with cortical areas, including those in the SN. For example, the AIC contains a high density of dopaminergic D_1_ receptors and receives strong dopaminergic inputs, and its signaling can be modified by dopamine [201,202,203]. In addition, the direction of influence can be reversed: the insula does appear to have some control over dopaminergic signaling. Transcranial magnetic stimulation targeted to the insula decreases dopamine levels in key reward circuitry, including the substantia nigra and striatum [204]. To directly probe the relationship between the SN and dopamine, McCutcheon and colleagues combined positron emission tomography (PET) to measure dopamine synthesis and release capacity with rs-fMRI in humans [205]. They showed that dopamine synthesis capacity (measured with ^18^F-DOPA PET) in limbic dopamine regions is associated with stronger connectivity strength within the SN, and this effect is fairly specific to the SN compared with other networks. Surprisingly, this effect is reversed for limbic dopamine release capacity (weaker SN connectivity associated with greater limbic dopamine release). One obvious functional link between the insula and dopamine is via the insula’s roles in interoception, which may add a unique contribution to how a substance is consumed and valued [206]. That is, not only salient external cues but also internal physiological and visceral sensations may be encompassed into the experience of reward through the insula [178,207].

## 4. Cross-Species Salience Network

The salience network was first identified in humans; however, there are limitations to the experimental studies that can be conducted with human subjects. Hence, the complementary use of nonhuman animals, such as rodents and monkeys, will be crucial to furthering our understanding of the SN and addiction. Thoughtful experimental designs can directly manipulate specific circuits to tease apart each component of the SN.

**Figure 1:**
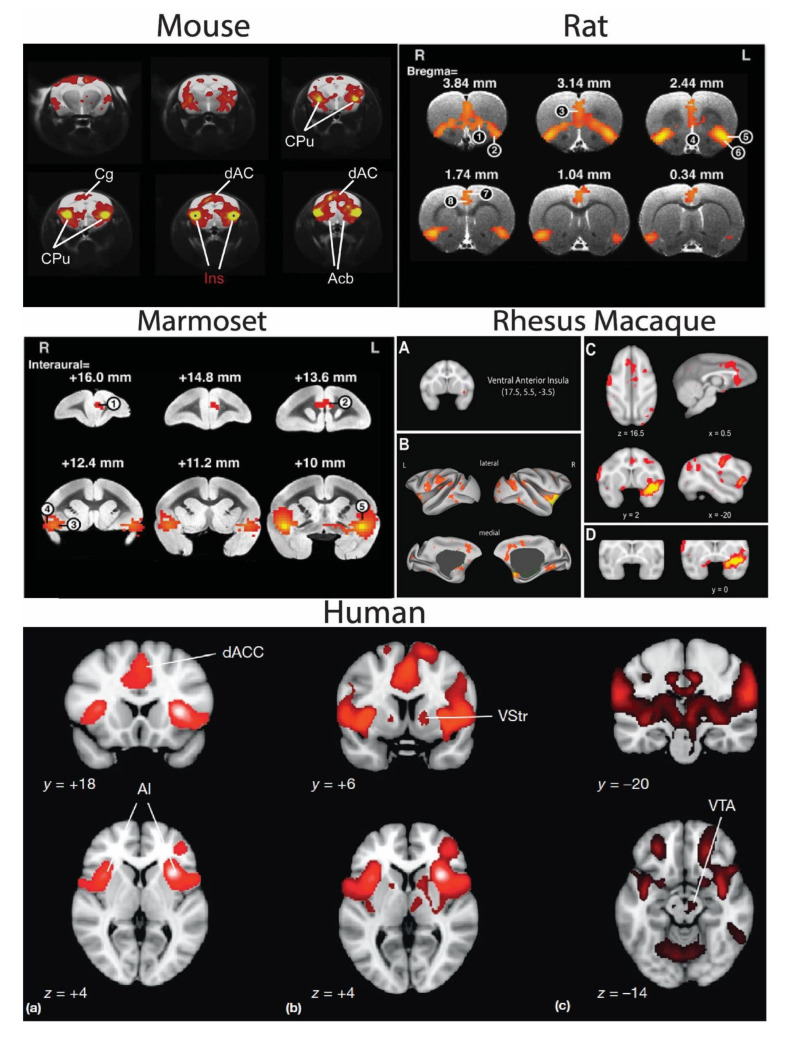
The SN of the mouse, rat, marmoset, rhesus macaque, and human. For all species, yellow indicates stronger functional connectivity. Reprinted with permission from Ref. [141]. 2020, Elsevier Reprinted with permission from Ref. [211]. 2014, Elsevier; Reprinted with permission from Ref. [36]. 2016, Elsevier; Reprinted with permission from Ref. [31]. 2016, Elsevier. **Mouse SN**: Bilateral seed in the insula revealed BOLD functional connectivity with the dACC and striatum. **Rat SN**: Seed region in the ventral AIC revealed functional connectivity with CG1(7), CG2(8), dorsal AIC (5), ventral AIC (6), ventral orbitofrontal cortex (1), lateral orbitofrontal cortex (2), prelimbic cortex (3), and infralimbic cortex (4). **Marmoset SN:** AIC connectivity with the medial prefrontal cortex (1), ACC (2), orbitofrontal cortex (3), gustatory cortex (4), and AIC (5). Thalamic connections were not noted (as in Belcher et al., 2013 [209]). **Rhesus Macaque**: (**A**) The ventral AIC seed is shown in the first panel; (**B**–**D**) display functionally connected regions—the dACC, subgenual cingulate, orbitofrontal cortex, amygdala, putamen, and temporal cortex (regions not previously observed in mouse, rat, and marmoset). **Human SN**: As described in the text, human SN includes the canonical AIC, dACC (**a**), ventral striatum (**b**), and ventral tegmental area (**c**). Abbreviations: CPu, caudate-putamen; Cg, cingulate cortex; dAC/ACC, dorsal anterior cingulate cortex; Acb, nucleus accumbens; Ins, insular cortex; vAI VO/LO, ventral/lateral orbital cortex; PrL, prelimbic cortex; IL, infralimbic cortex; AID/AIV, dorsal/ventral agranular insular cortex; Cg1/Cg2, primary/secondary cingulate cortex; mPFC, medial prefrontal cortex; OFC, orbitofrontal cortex; MCC, mid cingulate cortex; VTA, ventral tegmental area.

The SN has been described in mice, rats, marmosets, and rhesus macaques (Figure 1) [36,141,208,209,210,211]. Critically, all of the individual regions of the human SN appear to have homologues in these species, although some of the details of subregion designation may be controversial. For instance, there is some historical debate about whether nonhuman brains contain an AIC: insular primary sensory cortices appear to extend to the rostral edge of the macaque insula, perhaps leaving little room for a cognitive or emotional zone [42]. However, other authors treat the macaque AIC as homologous to the human AIC, with a conserved rostroventral to a dorsocaudal gradient of function, although perhaps the cognitive and emotional territories occupy proportionally less volume in macaques [70,103]. In marmosets, an orbital area that extends rostrally beyond the lateral sulcus has been proposed as a homologue to the human AIC [212,213]. However, there is some discrepancy about this region as Paxinos et al. 2012 classified this region as OPAI and OPro. Nevertheless, Reser and colleagues suggest that despite the difference in nomenclature, based on anatomical connectivity patterns (especially with the medial prefrontal cortex and AIC), this region in marmosets is likely to be the human AIC homologue [212,213,214].

The homology of the rodent AIC is drastically understudied, especially in mice with respective dorsal–ventral AIC divisions. However, a region of the mouse and rat brains appears cytoarchitectonically and connectionally similar to the AIC in primates [215,216,217]. Given that there are many possible criteria to establish homology [218], some of which are more challenging to assess across species, one possibility is that functional connectivity itself and the structure of the SN may provide valuable insights about AIC homology.

The dACC is also present in these species, but again, there is some question about the details. Humans have an extension of area 32 (called area 32′) in the dorsal portion of the dACC. The region of the dACC in macaque that is in a similar location, the dorsal bank of the cingulate sulcus, is a territory of some dispute, with some arguing that it is not cingulate cortex at all [119,219]. Still, both macaques and humans appear to have a dACC that contains rostral–caudal gradients of function and connectivity. For example, the rostral, middle, and caudal parts of the dACC receive dense anatomical inputs carrying affective, cognitive, and executive information, respectively, from the prefrontal cortex [114]. The marmoset brain also appears to contain an area 24 (dACC) caudal to area 32 [212]. Finally, in mice and rats, area CG (cingulate) is likely homologous to at least parts of the primate dACC [220,221]. However, in rats, striatal connectivity of area CG appears most similar to the connectivity of the caudal dACC, leaving the rostral dACC of primates potentially poorly represented in rodents [119]. In general, homologies of specific primate cortical regions can be challenging to ascribe to rodent brains; however, with the AIC and dACC, there is at least some evidence of homology across species.

What do we know about the SN’s presence/absence and organization in these nonhuman species? An essential property of intrinsic networks is that the activity is “bilateral and homotopic” [211]. Sforazzini and colleagues, using independent components analysis on resting-state fMRI data (BOLD and cerebral blood volume (CBV)) from anesthetized mice, identified an intrinsic SN meeting these criteria. They showed a network with bilateral AIC connectivity with the dACC and ventral striatum [211]. The seed region used in this study was a large anterior insular seed spanning both dorsal and ventral subregions, and it is unclear how a more restricted seed might affect the results (or not). Nevertheless, this network appears quite similar to the SN identified in humans.

Tsai and colleagues also demonstrated the presence of an SN in rats [141]. Using a seed-based analysis on resting-state fMRI data from anesthetized animals, they showed that the ventral anterior insular cortex was functionally connected with the rest of the AIC and CG (the likely homologue of primate dACC). However, there were also many other frontal regions involved. A similar (though not identical) network structure was derived from anatomical tract-tracing data. This group then directly compared the SN of rats, marmosets, and humans by examining the resting-state functional connectivity of a ventral AIC seed. Again, there were many commonalities across the three species, including connectivity with the dACC and the broader AIC. However, using this analysis, only the human SN included the striatum. The marmoset and rat SNs, by contrast, were the only ones that included the orbitofrontal cortex [141].

Touroutoglou and colleagues detailed a homologous SN in the rhesus macaque [36]. A seed-based analysis similar to an earlier study [208] concluded that nonhuman primates also have an intrinsic SN anchored in the ventral agranular insular cortex [36]. In addition, the ventral agranular insular cortex displayed intrinsic connectivity with the dACC, orbitofrontal cortex, amygdala, putamen, and fronto-insular cortex [36]. However, unlike the human SN, the nonhuman primate SN displayed additional connectivity with superior temporal and frontal regions [208]. According to this study, macaques do not possess what is termed the ‘dorsal salience network,’ which, as described above, is probably what would now be considered the CON [36]

Notably, anatomical projections between the core nodes of the SN and dACC have been established in nonhuman animals, particularly through the tract-tracing studies mentioned above (e.g., [63,64]). These findings have been commonly cited as evidence that functional interactions within the SN arise from direct anatomical connections [55]. However, the observation of human dACC–AIC connections with dMRI tractography remains scarce [75]. Establishing a cross-species homology is important for probing SN functions in animal models and translating the ground-truth knowledge from tract-tracing studies into human neuroanatomy to guide dMRI tractography investigations [68,130].

### Circuits/Manipulations of the SN

Studies of the SN in humans have been important in establishing the network, identifying many of its cognitive and emotional functions, and determining disorders in which it is impacted. However, human studies have inherent limitations relative to nonhuman animal work. Molecular and genetic tools have been developed for use in nonhuman animals that are simply not suitable for human subjects. These tools allow for the dissection of the specific circuit components that are important to different characteristics of the SN. Chiefly, we are concerned below with the application of optogenetics and chemogenetics to the SN. In brief, optogenetics uses genetically coded light-driven ion channels and pumps (opsins) that can excite or inhibit neuronal activity [222,223,224,225]. Chemogenetics uses genetically modified G-protein coupled receptors—designer receptors exclusively activated by designer drugs (DREADDs)—that can also inhibit or excite cellular activity depending on the exogenous receptor type [226,227,228,229]. Once expressed, DREADDs are activated by the binding of an actuator ligand [230,231,232,233]. Optogenetics has excellent temporal resolution, which can rapidly activate or deactivate cells, whereas DREADD-induced effects are typically slower but last for a longer period of time [234,235,236,237]. Nevertheless, both techniques allow for studying specific cell types and projections in the SN.

Both optogenetics and chemogenetics have been applied chiefly in mice, but also in rats, marmosets, and macaques. However, because of the uncertain homologies discussed above, it is worth noting that there are unique challenges associated with performing these methods outside of mice, and especially when performing them in nonhuman primates. In mice (and sometimes in rats but rarely in marmosets), circuit specificity can be achieved by developing a transgenic line of animals [222,228,238,239,240]. In other species (especially macaques), viral approaches are needed [224,241,242,243]. Viral vectors can be designed such that the target opsin or DREADD is encoded and delivered to the target brain region or circuit. Additional circuit specificity can be achieved via intracranial infusion of ligand [244], targeted light delivery [245,246,247,248], or via an intersectional viral approach (such as the Cre-DIO system). However, outside of mice, there is reduced capability to encode for specific cell types, and factors such as the virus being used, the carrying capacity, and the size of the genetic material being delivered must also be considered [249,250]. Furthermore, different virus serotypes can interact differentially with the target region’s cellular composition to alter the expression of opsins or DREADDs [251]. Another non-negligible challenge is the sheer difference in brain sizes across species [224]. Hence, in nonhuman primates, a larger volume of tissue has to be targeted. Importantly, this has to be performed in a manner that will yield high expression without causing significant damage to the tissue.

With these caveats in mind and having established that the SN is present in these nonhuman animal model species (even if it may not be completely identical to the human SN), it is possible to utilize molecular and genetic tools to dissect the circuit underpinnings of the SN. The activity of the SN affects not only the dynamics among its nodes but also the dynamics of other closely linked networks, particularly the DMN and CEN.

Homologues of each of the three components of the triple network were previously known to exist in nonhuman brains [26,32,35]; however, whether the individual networks combine in a manner that is similar to the triple network identified in humans was unknown [26,36,141,211].

To investigate the triple network framework in nonhuman animals, Mandino and colleagues combined many methods (awake and resting-state fMRI datasets, optogenetic neuromodulation, and viral tract tracing) and multiple species (humans, macaques, and mice) [35]. Rs-fMRI data were decomposed into functional networks for mice, macaques, and humans. Qualitatively, the CEN, DMN, and SN were represented in all three species [35]. This reinforced that the triple network is not only a feature of humans but also macaque and mouse brains.

One open question is whether the features of specific psychiatric disorders can be recapitulated in nonhuman animals. Indeed, in a rodent model of depression induced through chronic social stress [252,253], the DMN was hyperactive, and the SN was hypoactive, mirroring results in humans [35]. Thus, not only were cross-network features recapitulated, but the aberrant underlying networks were also consistent across models. This property makes it feasible to study these aberrant activities in nonhuman models.

Mandino et al., (2022) then went one step further, using the molecular tools available in rodents to probe the biological underpinnings of the triple network. First, they injected viral tract tracers into nodes of the three networks to assess anatomical connectivity and found that these nodes received projections from mainly non-overlapping upstream regions. In other words, the DMN, SN, and CEN receive most of their inputs (in mice) from different brain regions. Next, using optogenetics, they activated the CamKII-positive insular neurons and dorsal raphe ePet-positive (serotonergic) neurons while performing fMRI. Both resulted in activation patterns consistent with the SN, emphasizing the potential role of the insula and dorsal raphe serotonergic neurons in this network. Although the link between the SN and serotonin was, in the words of the authors, “unexpected,” there is evidence of serotonin’s involvement in other networks, particularly the DMN [254,255], and these findings highlight the importance combining molecular methods with fMRI. Finally, although stimulation of insular neurons did not lead to positive conditioned place preference, the extensive inter-subject variability in behavioral response was associated with differential network engagement across the SN and DMN.

To directly probe the role of the AIC in modulating other networks, Menon and colleagues also combined optogenetic stimulation and rs-fMRI, although in rats [256]. Optogenetic stimulation of the AIC increased both AIC activity and functional connectivity with other regions of the SN [45,102,256,257]. However, the effects of AIC stimulation were not limited to the SN. Stimulation also suppressed activity in the DMN, reduced functional connectivity within the DMN, and reduced functional connectivity between the SN and the DMN. This result suggests that the AIC has an influential inhibitory input to the DMN and may be key to the dynamic relationships among the DMN, CEN (although the CEN was not addressed here), and SN. Moreover, this study directly assessed and proposed a circuit mechanism that could facilitate the dynamic switch in activity—DMN inactivation and concurrent activation of the SN—observed in typical brain functioning and altered in addiction disorders. They also note that the DMN hub in rodents (retrosplenial cortex) does not receive a direct projection from the AIC and suggest that the CG, prelimbic cortex, and/or claustrum may be acting as intermediaries in this circuit. This represents a crucial mechanistic insight into the triple network model, although future work will be needed to understand not just how the SN may control the DMN, but also how it may control the balance between the DMN and CEN.

To investigate the electrophysiological basis of the SN and DMN, Chao and colleagues recently combined fMRI with simultaneous fiber photometry recording of neuronal activity (via GCaMP, a calcium indicator) in awake and resting rats [258]. GCaMP recordings revealed significant functional connectivity among and between nodes of the DMN (retrosplenial cortex and medial prefrontal cortex) and the SN (AIC and medial prefrontal cortex), indicating that the pattern of functional connectivity canonically identified using fMRI is also present in low-power spectral power fluctuations of GCaMP signals. GCaMP changes also preceded network-level activations and deactivations in the DMN and SN. Critically, similar to Menon et al., (2023), Chao et al., (2023) found that AIC had an inhibitory effect on the retrosplenial cortex and medial prefrontal cortex, and thus, on the DMN [256,258]. Furthermore, an auditory oddball paradigm resulted in an increase in GCaMP activity in the AIC, but a decrease in DMN areas, consistent with prior findings in human fMRI [45].

The dACC is a crucial region in the human SN. However, it is also a highly connected central hub region of the brain, such that the ACC facilitates communication across and between many brain regions. Accordingly, Peeters and colleagues [259] investigated how inhibition of CG (the rodent dACC homologue) activity could alter communication in the brain. Following unilateral designer receptors exclusively activated by designer drugs (DREADDs) expression in the CG, functional connectivity changes were assessed with fMRI. CG inhibition increased activity in the retrosplenial cortex, insular cortex, and basolateral amygdala. This was coupled with a simultaneous decrease in activity in the visual cortex and thalamus [259].

The medial prefrontal cortex in the rodent is a large region that likely contains nodes of multiple networks. Rocchi et al., (2022) targeted the infralimbic, prelimbic, and anterior cingulate cortices in mice, likely involving, at a minimum, the DMN and SN [260]. Specifically, by using transgenic rodents, researchers explored the effects of acute and chronic inhibition on network organization and dynamics. The medial prefrontal cortex was studied under conditions of chronic and acute inhibition. The literature on functional connectivity suggests that altering the activity of a critical brain region within a network would result in reduced functional coupling between the target region(s) and other brain regions that received direct anatomical projections from the target [260,261,262,263]. Surprisingly, rsfMRI showed that chronic and acute inhibition of the medial prefrontal cortex resulted in hyperconnectivity of midline structures such as the posterior cingulate cortex and mediodorsal thalamus. This study highlights the complex interplay between direct anatomical connectivity and functional connectivity. One possible explanation is that the involvement of multiple competing networks may explain the counterintuitive results.

There is a vast literature on specific neural circuit and cellular mechanisms that contribute to addiction-like processes in nonhuman animals [200,200,264,265]. Together with the human clinical literature (reviewed above), these studies highlight the role of the dACC in drug-related disruptions in inhibitory control and the role of the AIC in interoception, craving, and relapse (e.g., [147,266,267,268,269,270]). A thorough review of this expansive literature is beyond the scope of this paper. However, generally missing is an approach that evaluates all of the regions of the SN, as well as other relevant networks. Nevertheless, very frequently, key SN regions are highlighted. For example, chemogenetic stimulation of the rat AIC reduced alcohol consumption and enhanced cerebral blood volume in both the AIC and the medial prefrontal cortex, which is suggestive of possible SN involvement [271].

## 5. Conclusions

The SN interplay with addiction is becoming more evident as researchers actively explore the dynamics between anatomical connectivity, functional brain networks, the brain regions that comprise these networks, and the aberrations that underlie and support addiction. Functional networks are not only a feature of the human brain, but homologous brain states and functional properties are also present in nonhuman animals commonly used in research. Furthermore, while the aberrant network activity identified in human addiction studies has not been directly replicated in nonhuman animal models, the discovery of homologous regions and networks and the successful recapitulation of other brain disorders is promising on this front. Moreover, although not the focus of this review, there are extensive studies on how individual nodes of the SN are involved in specific addiction processes in nonhuman animals [272,273,274]. This information can help to guide future mechanistic network studies. Finally, both the AIC and the ACC are the subjects of extensive functional study in humans and nonhuman animals, separate from examinations of the SN. Such studies use promising computational approaches to evaluate these regions’ roles in decision-making, affect, and cognition. Just as we expect circuit and network approaches to complement one another as they move towards common explanations, we expect computational, region-specific studies of the AIC and ACC to inform our understanding of the SN.

## Data Availability

Not applicable.
